# Supplementary material for: Multiomic Strategies Reveal Diversity and Important Functional Aspects of Human Gut Microbiome
Source: Biomed Res Int. 2018 Nov 14;2018:6074918. doi: 10.1155/2018/6074918 (PMC6280293; doi:10.1155/2018/6074918)
Supplement: Supplementary Materials — Supplementary Figure 1. Fecal metatranscriptome library preparation. High-quality total RNA from a fecal sample was isolated and analyzed by agarose gel electrophoresis and Bioanalyzer (A); total RNA was enriched for mRNA by depleting the rRNA by subtractive hybridization method (B); the enriched mRNA was fragmented by Covaris (C); a library was prepared using Illumina compatible adaptor (D); in addition, 12 libraries from the same mRNA were prepared for multiplexing (E). The quality of RNA, mRNA, and the libraries was analyzed on 2100 Bioanalyzer Instrument. Supplementary Figure 2. Phylum level analysis of multiplexed libraries using read and contig based analysis. The twelve metatranscriptome libraries were sequenced on Illumina MiSeq (151 PE) and analyzed for bacterial taxonomic assignment at phylum level using sequence read (A) and assembled contigs (B). Also, all the twelve libraries were combined in silico and called Lib-all. Supplementary Figure 3. Genus level analysis of multiplexed libraries using read and contig based analysis. The twelve metatranscriptome libraries were sequenced on Illumina MiSeq (151 PE) and analyzed for bacterial taxonomic assignment at genus level using sequence read (A) and assembled contigs (B). Also, all the twelve libraries were combined in silico and called Lib-all, and top 1% genus are shown (data sorted high to low abundance in Lib-all). Supplementary Figure 4. Species level analysis of multiplexed libraries using read and contig based analysis. The twelve metatranscriptome libraries were sequenced on Illumina MiSeq (151 PE) and analyzed for bacterial taxonomic assignment at species level using sequence read (A) and assembled contigs (B). Also, all the twelve libraries were combined in silico and called Lib-all, and top 1% bacterial species are shown (data sorted high to low abundance in Lib-all). Supplementary Figure 5. Taxonomic analysis of the metatranscriptome. The read and contig based analysis of HS100, MS151, MS301, and [file 6074918.f1.pdf]

Supplementary Figure 1

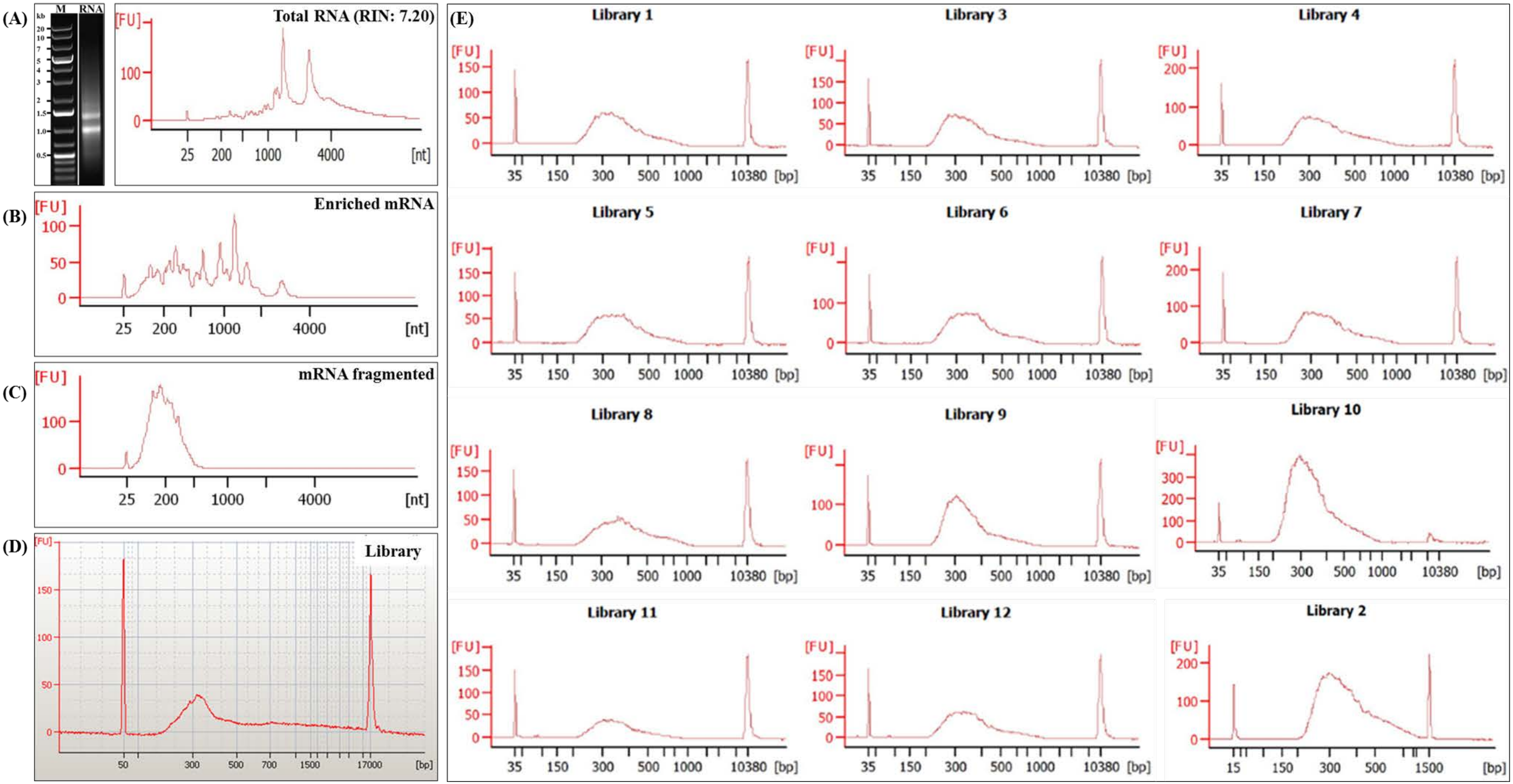

Supplementary Figure 2

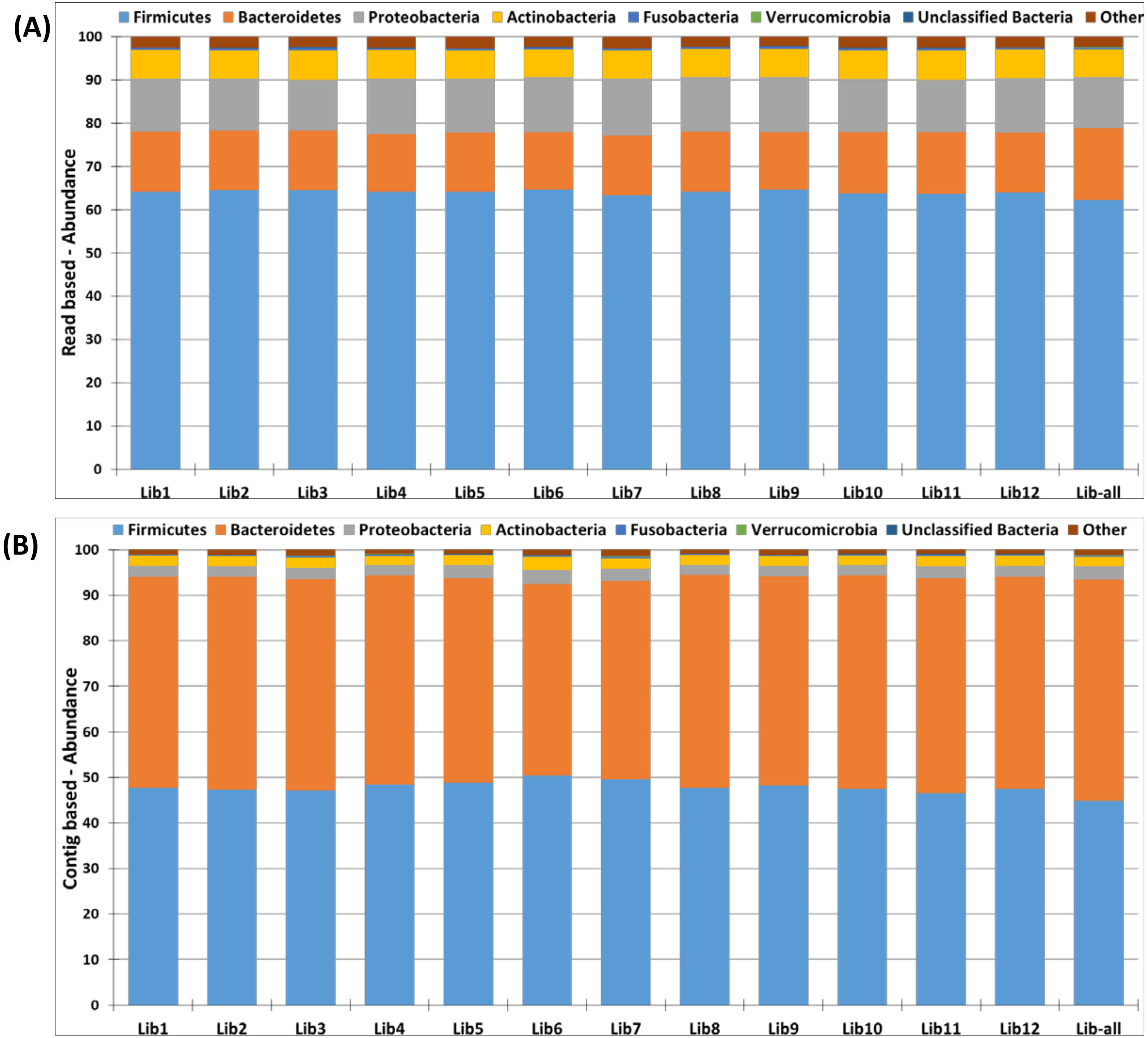

Supplementary Figure 3

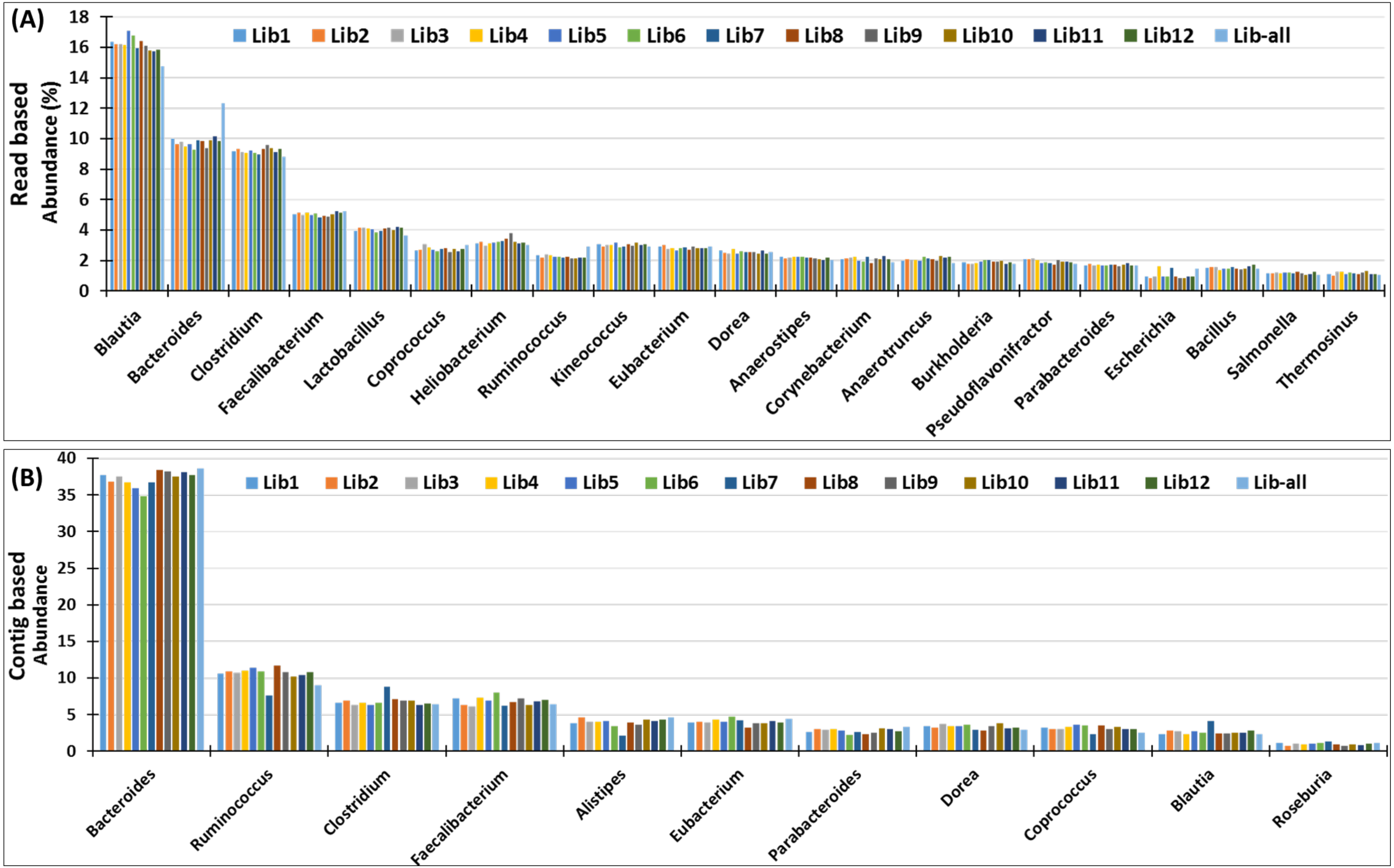

Supplementary Figure 4

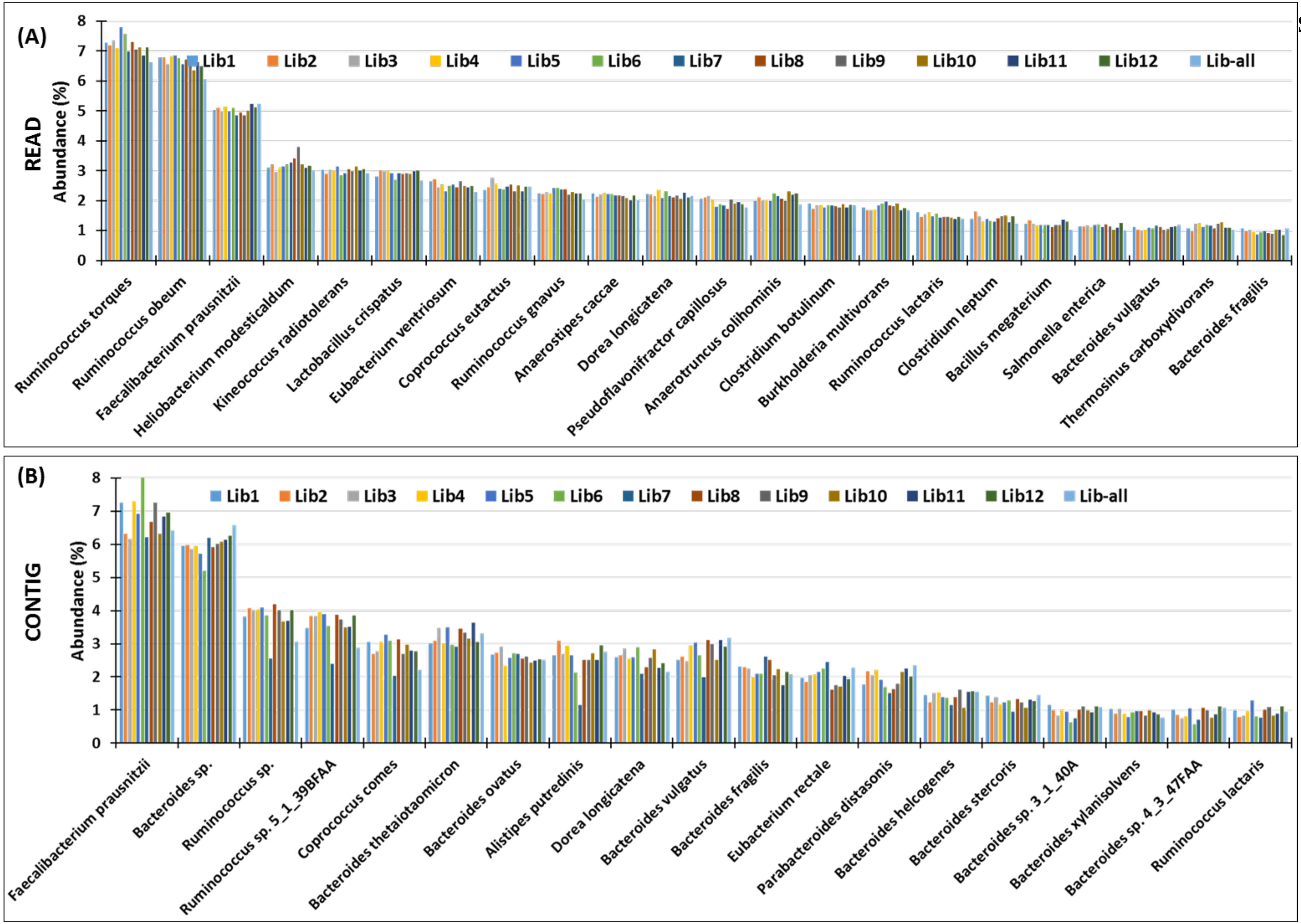

Supplementary Figure 5

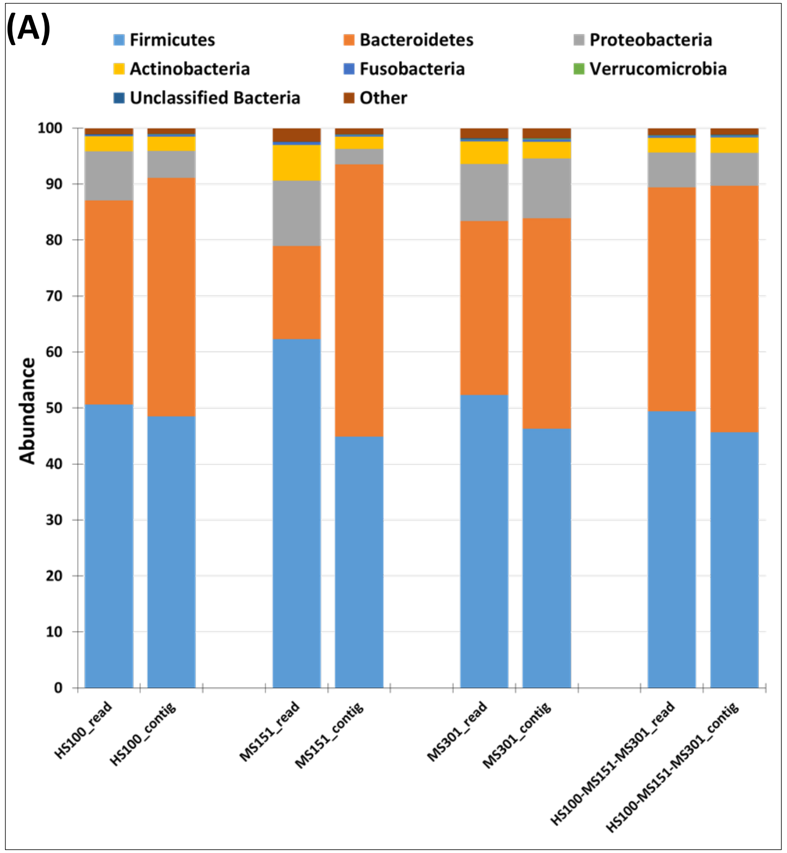

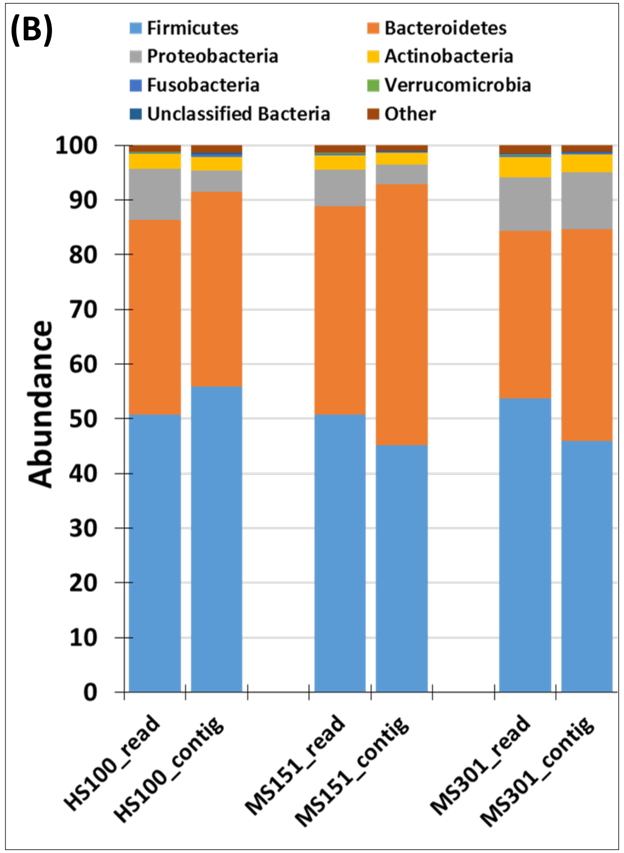

Supplementary Figure 6

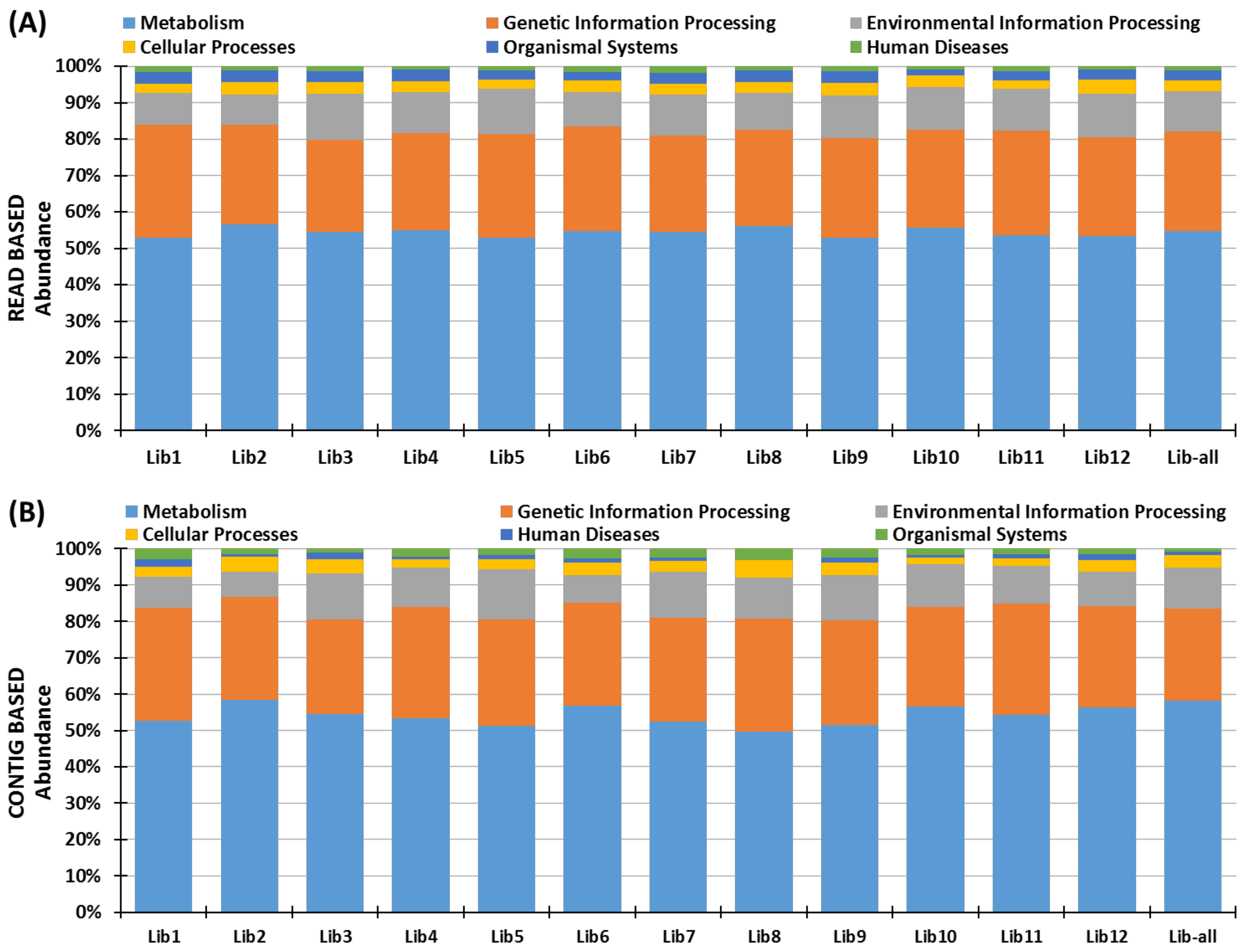

Supplementary Figure 7

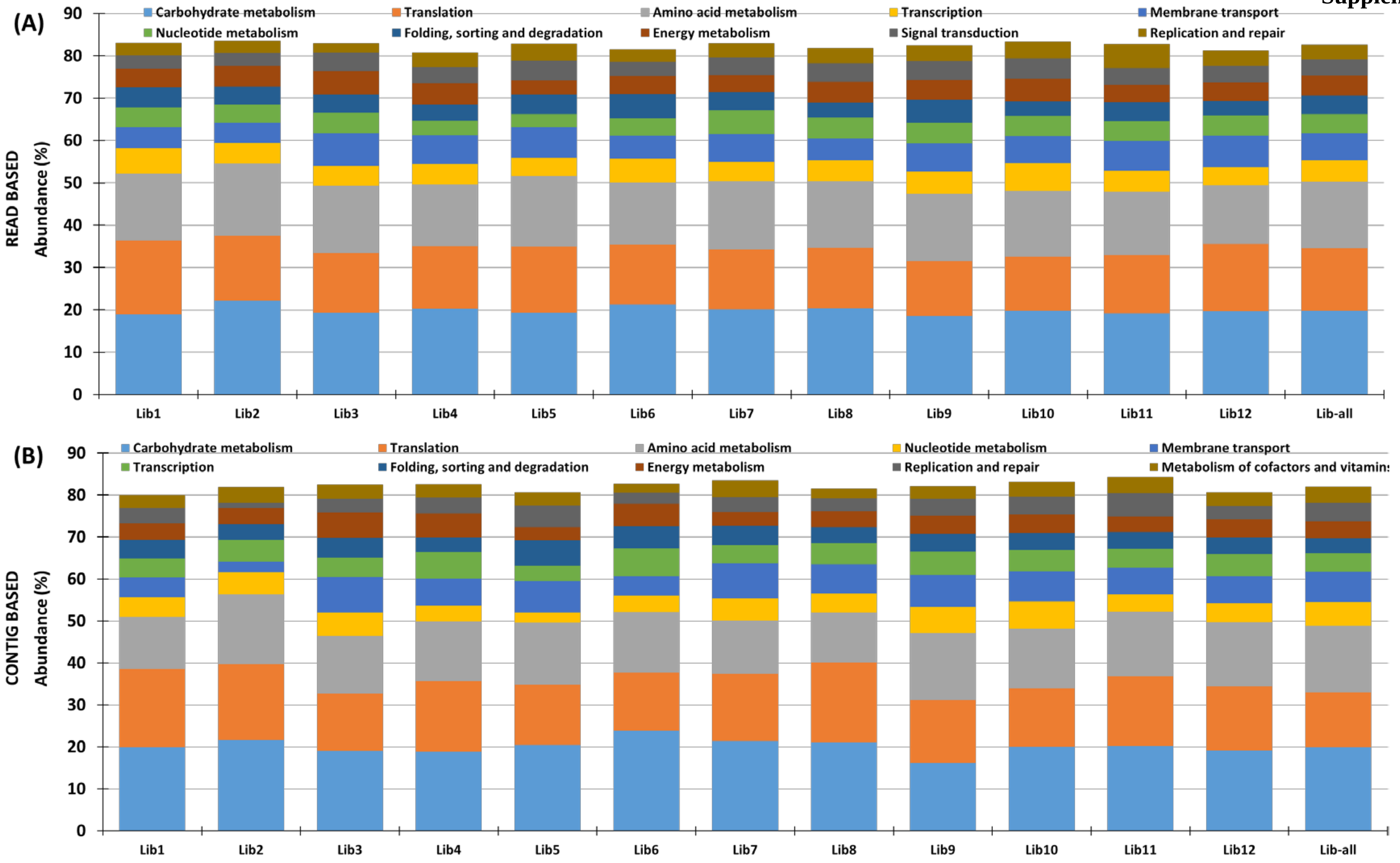

Supplementary Figure 8

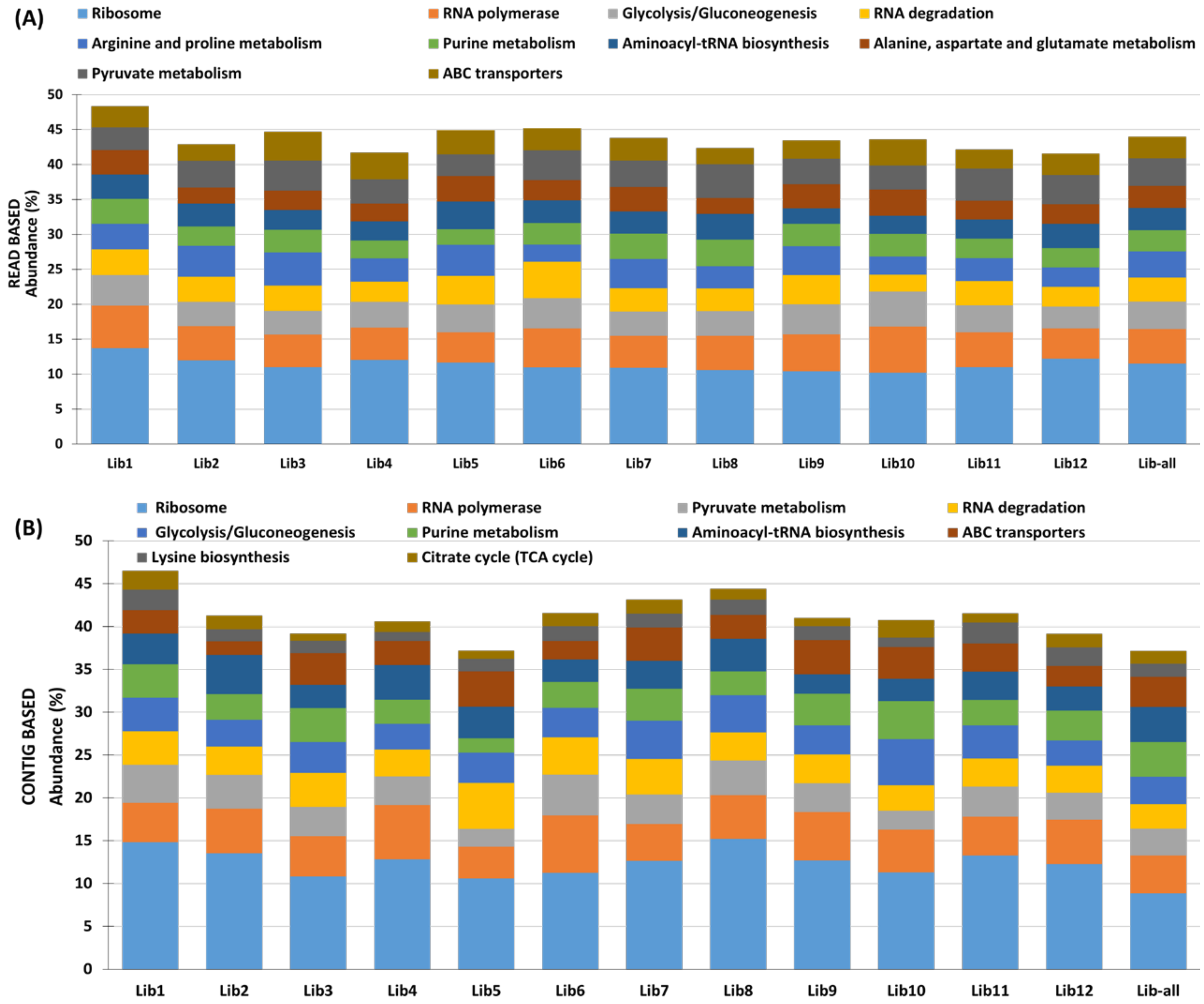

Supplementary Figure 9

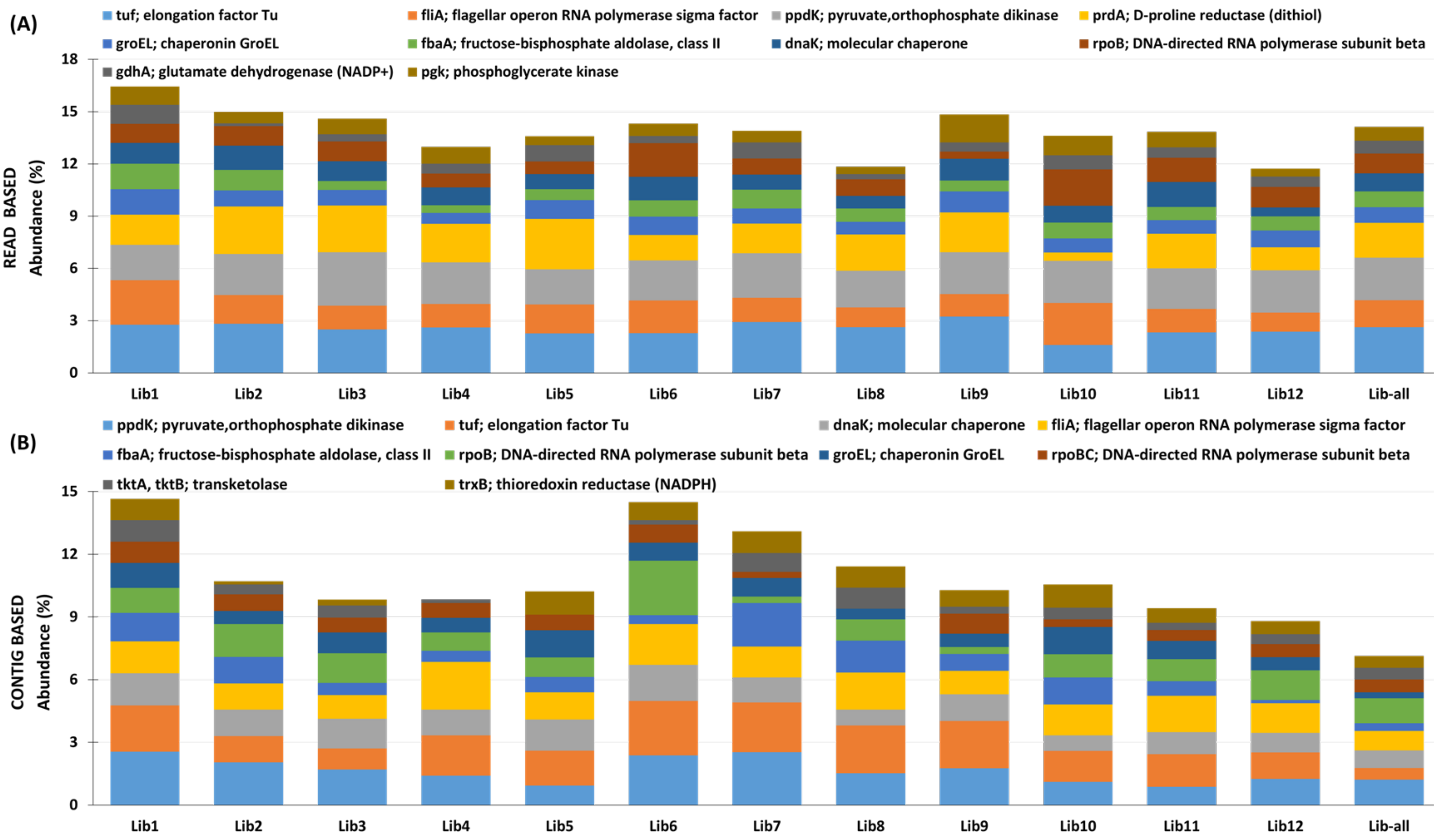

Supplementary Figure 10

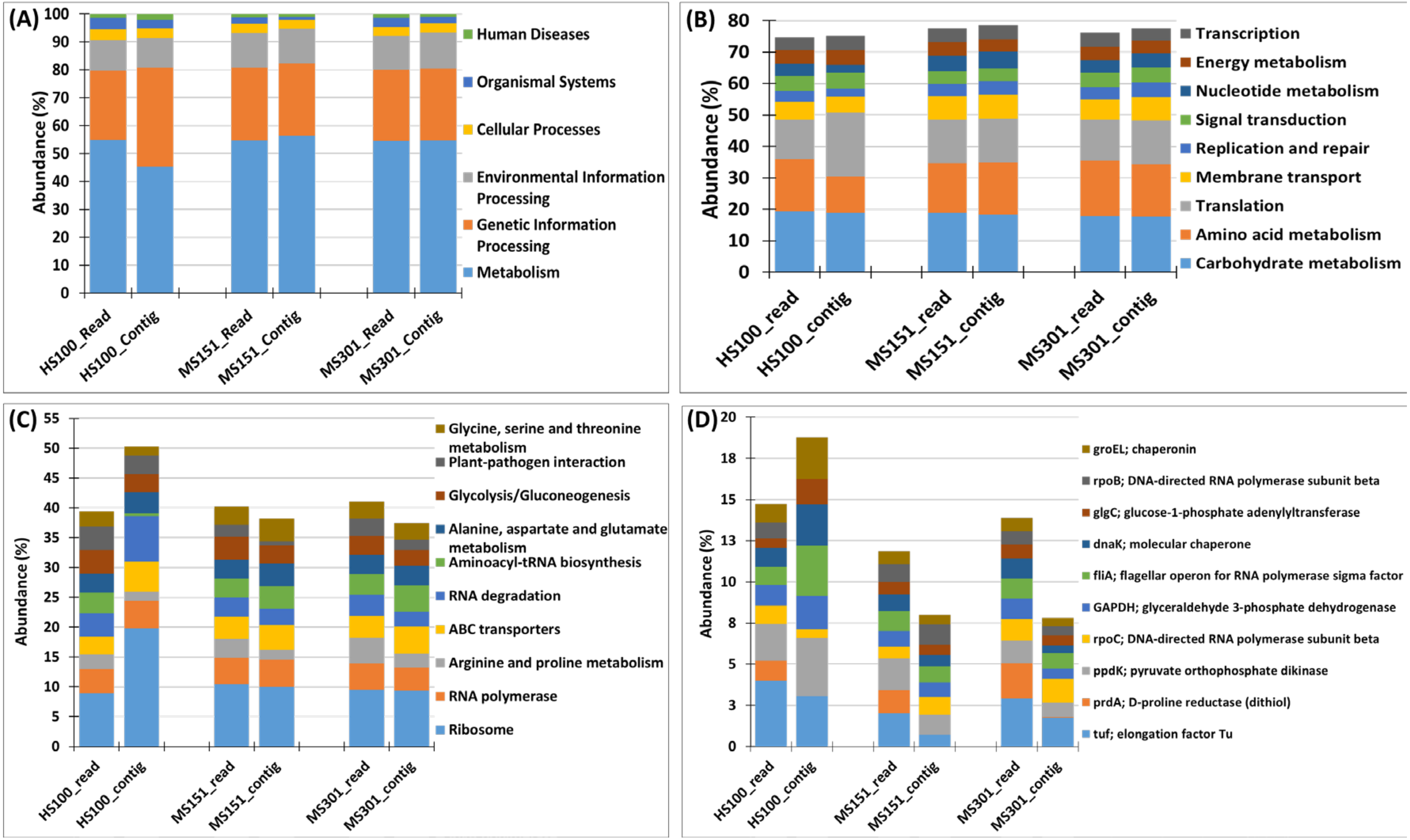

Supplementary Figure 11

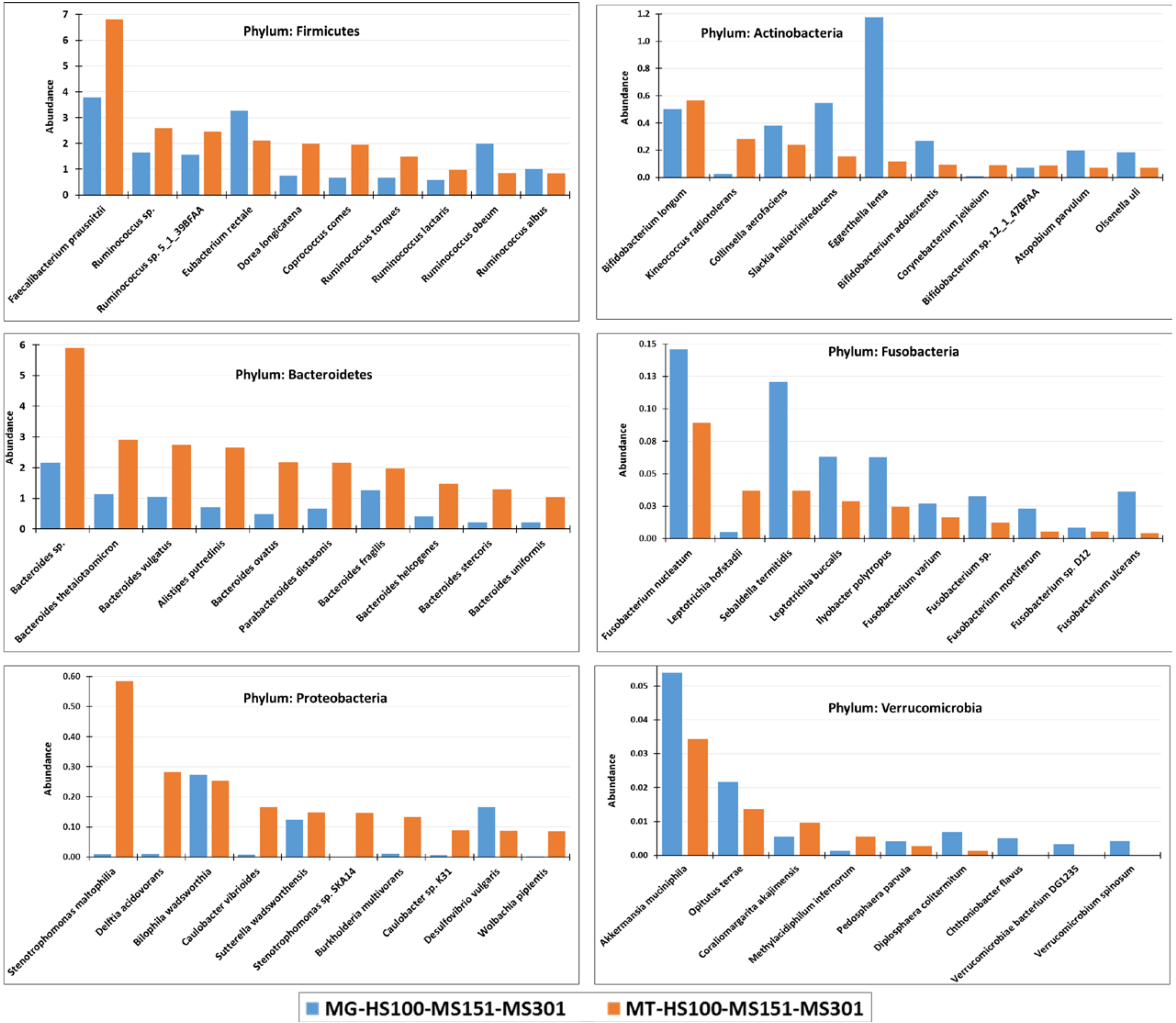

Supplementary Figure 12

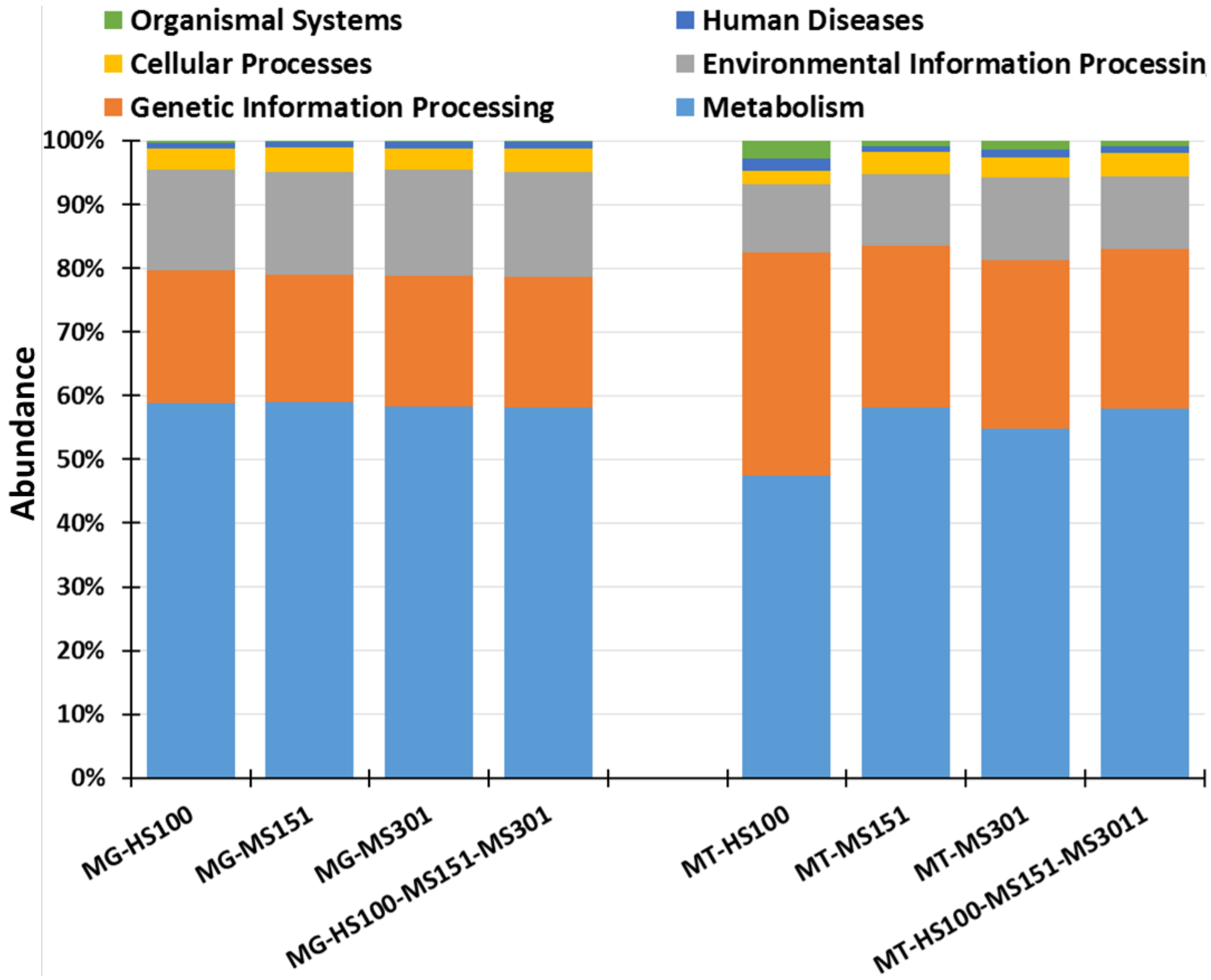

Supplementary Figure 13

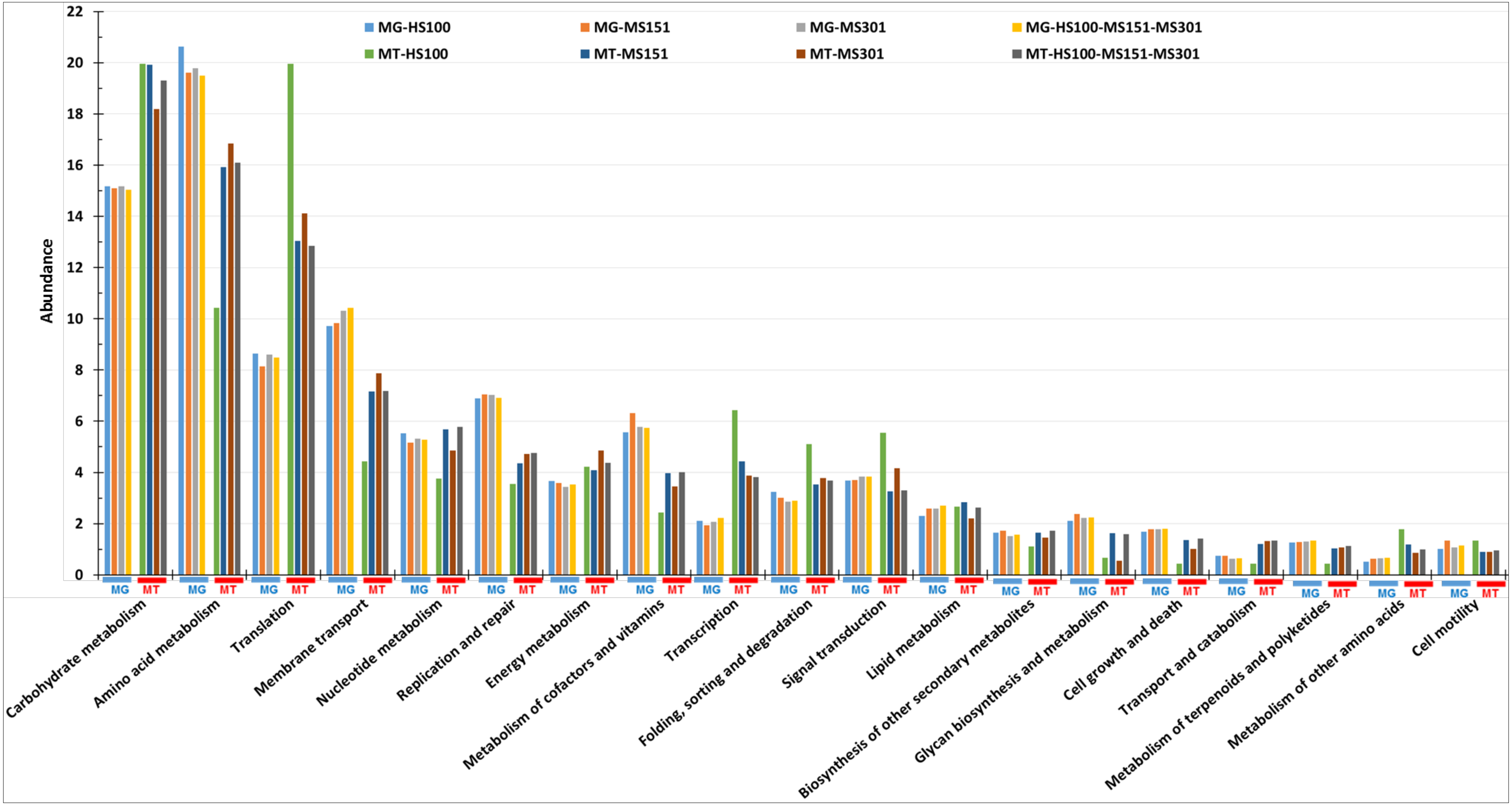

Supplementary Figure 14

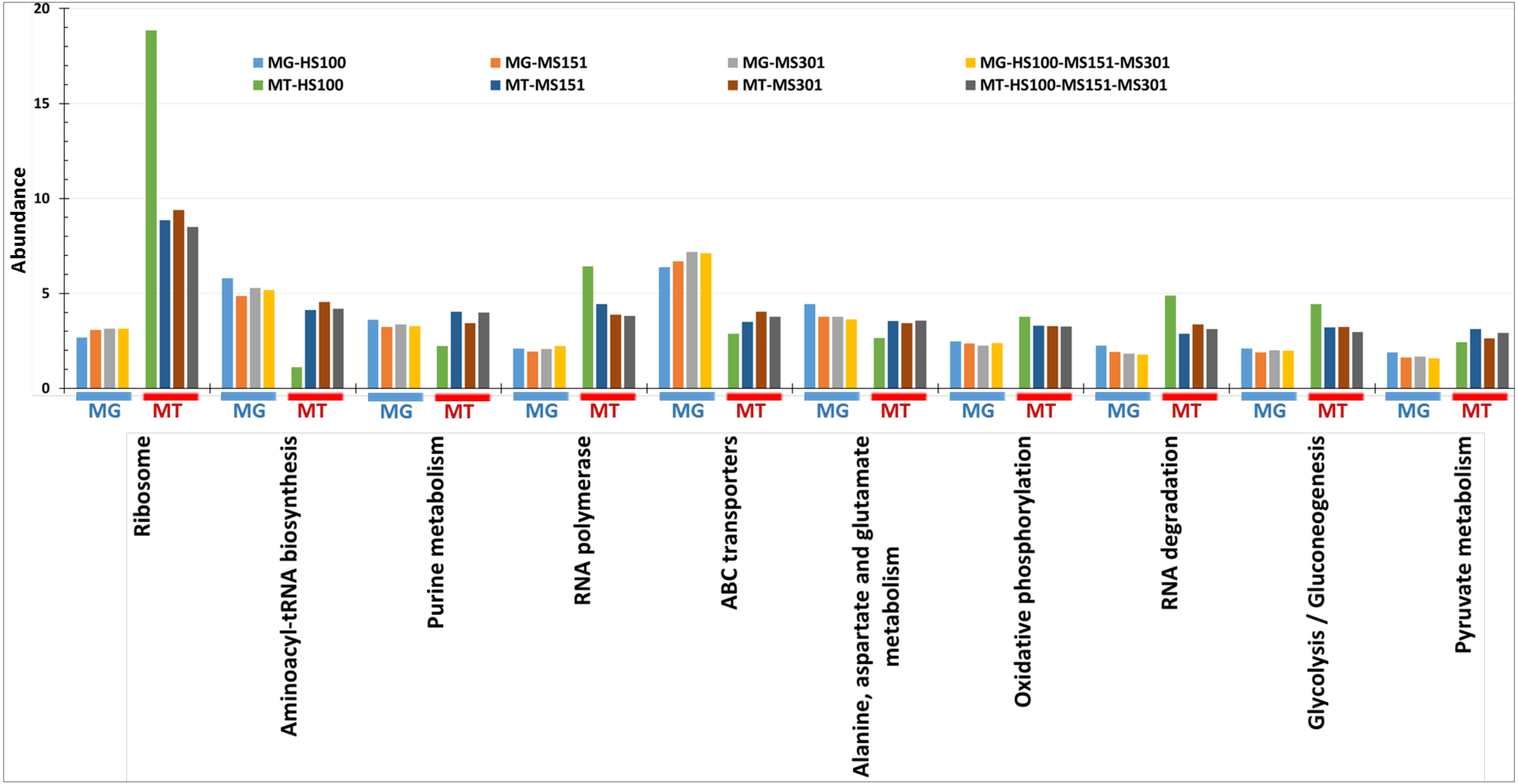

Supplementary Figure 15

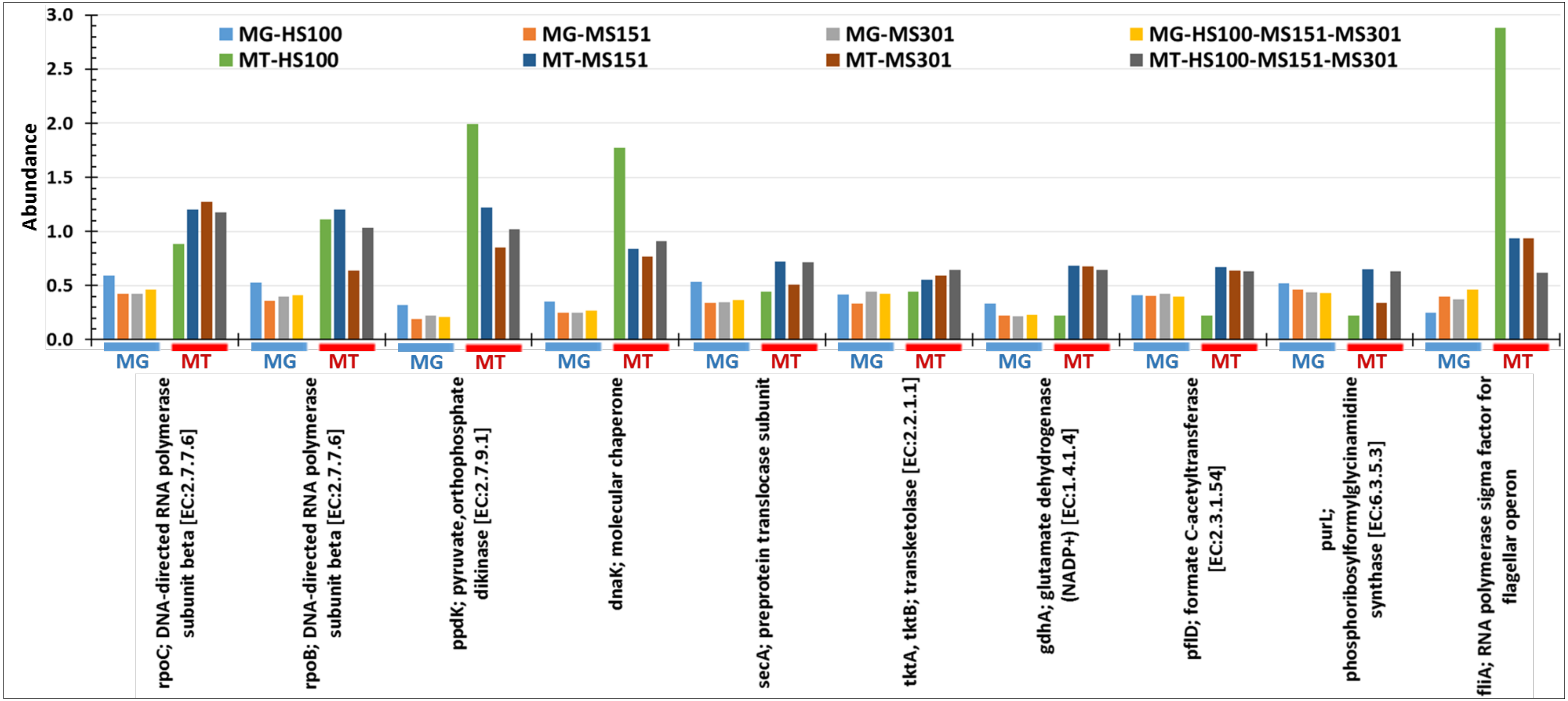

Supplementary Figure 16

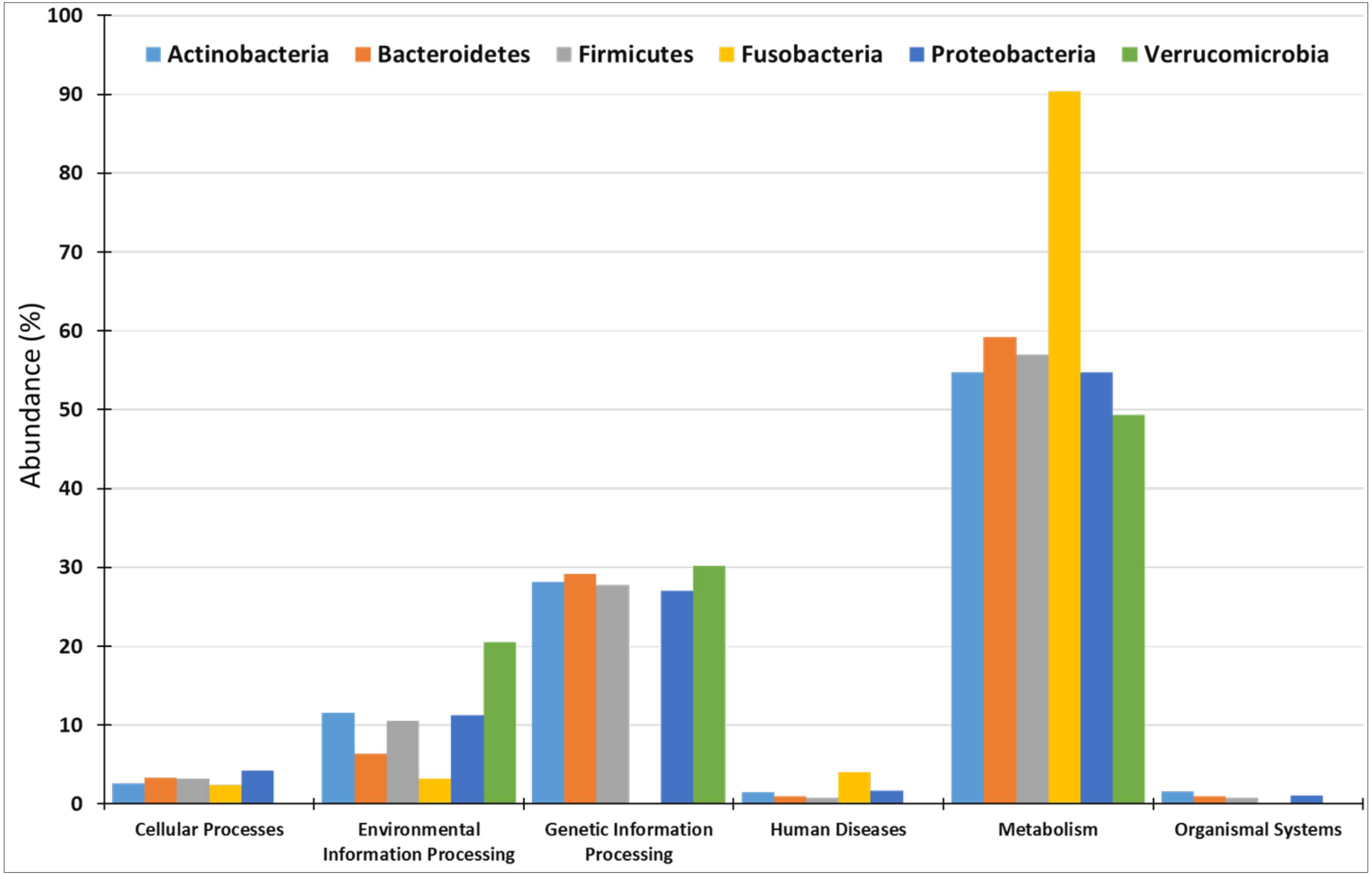

Supplementary Figure 17

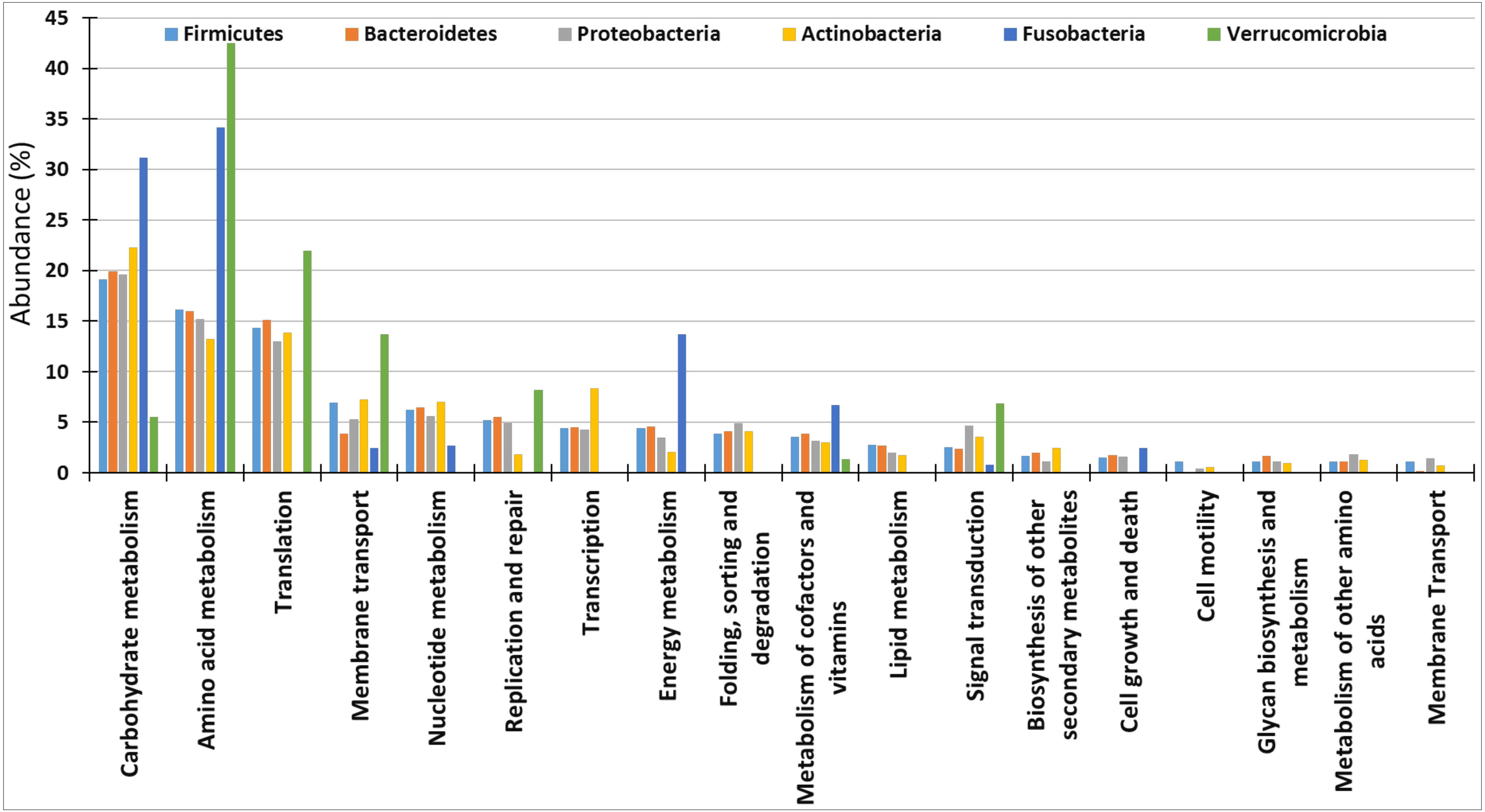

Supplementary Figure 18

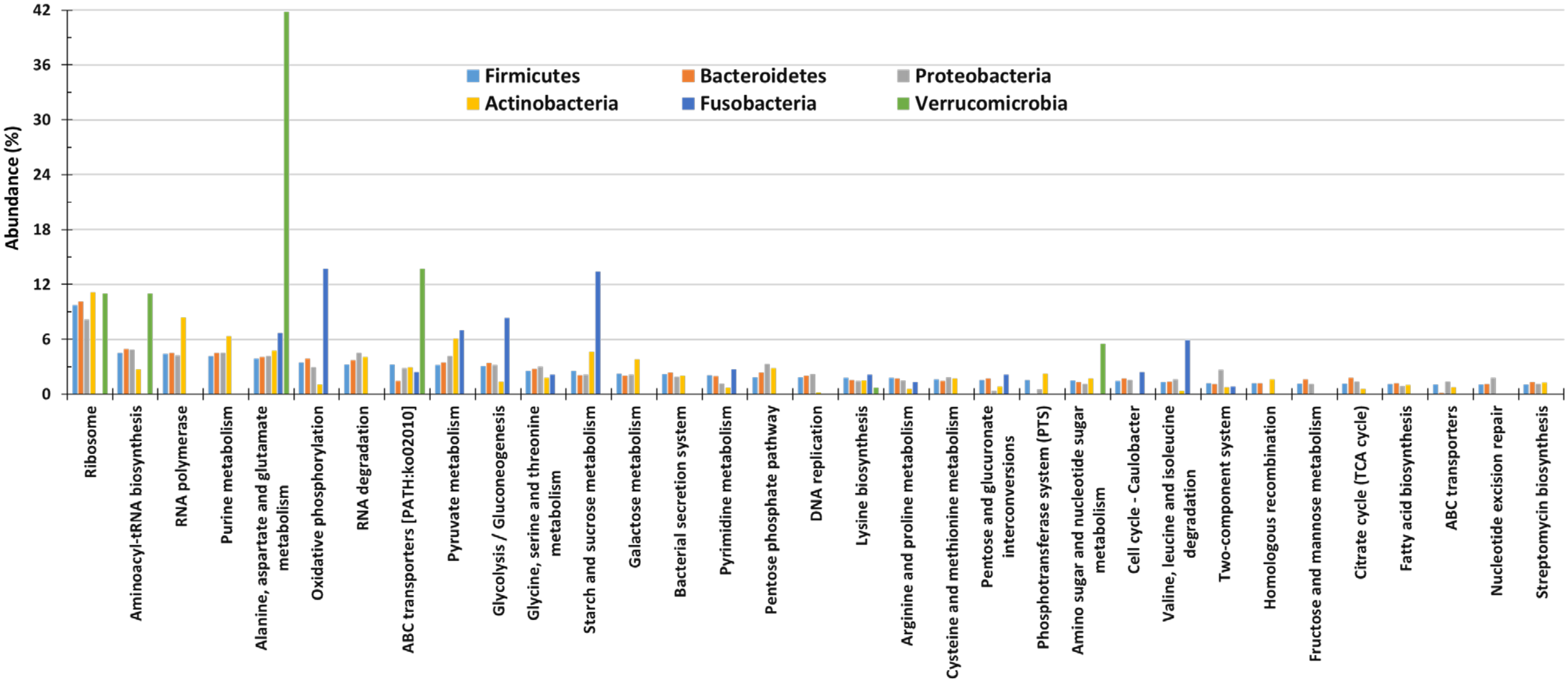

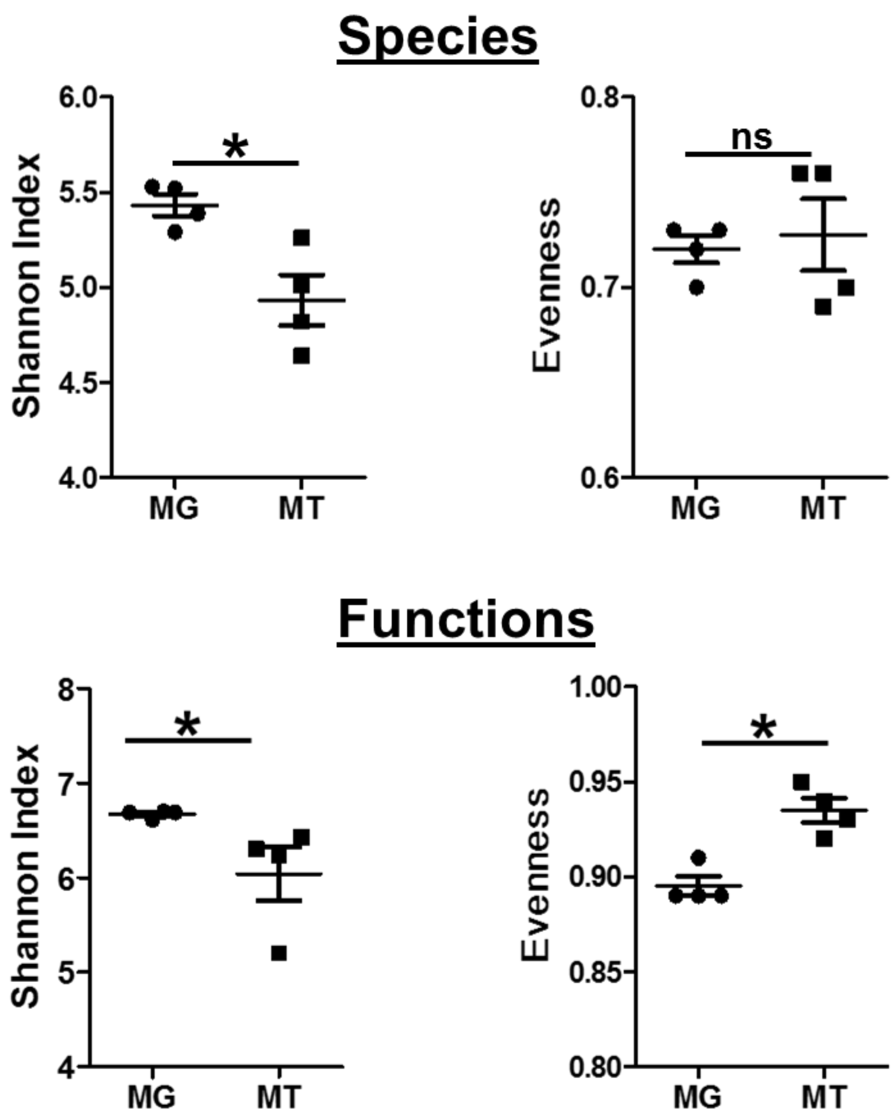
Species
6.0
5.5
5.0
4.5
4.0
Shannon Index
*
MG
MT
0.8
0.7
0.6
Evenness
ns
MG
MT
Functions
8
7
6
5
4
Shannon Index
*
MG
MT
1.00
0.95
0.90
0.85
0.80
Evenness
*
MG
MT

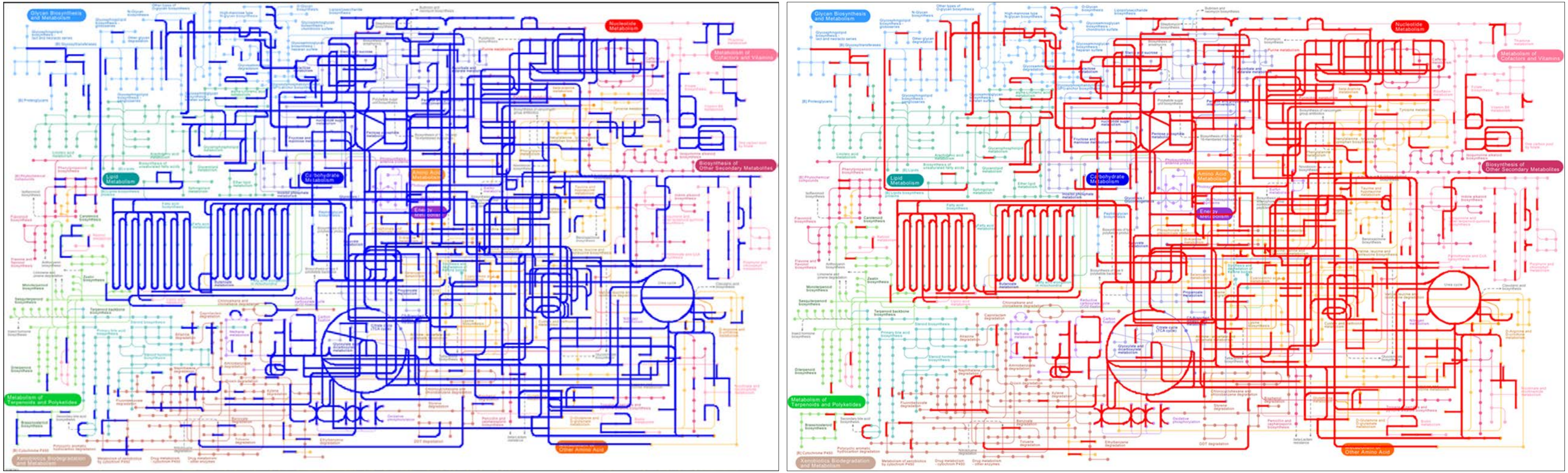

Blue: Exclusive in metagenome; Red: Exclusive in metatranscriptome

Supplementary Table 1. List of bacterial species identified based on read based analysis. Only above 1% are mentioned and sorted high to low on Lib1.

Phylum	Species	Lib1	Lib2	Lib3	Lib4	Lib5	Lib6	Lib7	Lib8	Lib9	Lib10	Lib11	Lib12	Lib-all
Firmicutes	*Ruminococcus torques*	7.29	7.19	7.34	7.11	7.79	7.57	6.99	7.31	7.05	7.12	6.86	7.12	6.63
Firmicutes	*Ruminococcus obeum*	6.79	6.78	6.55	6.82	6.86	6.76	6.57	6.72	6.82	6.36	6.63	6.49	6.07
Firmicutes	*Faecalibacterium prausnitzii*	5.03	5.11	4.98	5.14	4.98	5.11	4.84	4.93	4.86	5.01	5.24	5.13	5.24
Firmicutes	*Heliobacterium modesticaldum*	3.10	3.21	2.97	3.10	3.15	3.20	3.27	3.41	3.79	3.21	3.09	3.17	3.01
Actinobacteria	*Kineococcus radiotolerans*	3.04	2.89	3.03	3.00	3.14	2.85	2.92	3.05	2.97	3.14	3.00	3.06	2.92
Firmicutes	*Lactobacillus crispatus*	2.79	3.00	2.99	2.99	2.91	2.70	2.92	2.89	2.92	2.89	2.97	3.01	2.66
Firmicutes	*Eubacterium ventriosum*	2.63	2.71	2.45	2.52	2.31	2.50	2.53	2.44	2.64	2.49	2.45	2.50	2.29
Firmicutes	*Coprococcus eutactus*	2.35	2.45	2.76	2.57	2.40	2.38	2.47	2.54	2.31	2.50	2.30	2.46	2.46
Firmicutes	*Ruminococcus gnavus*	2.25	2.22	2.28	2.23	2.43	2.42	2.37	2.36	2.20	2.29	2.23	2.23	2.04
Firmicutes	*Anaerostipes caccae*	2.25	2.13	2.20	2.26	2.21	2.22	2.18	2.18	2.15	2.08	2.02	2.18	2.01
Firmicutes	*Dorea longicatena*	2.22	2.20	2.16	2.35	2.07	2.30	2.15	2.11	2.17	2.06	2.26	2.11	2.15
Firmicutes	*Pseudoflavonifractor capillosus*	2.06	2.11	2.15	2.05	1.80	1.88	1.83	1.72	2.03	1.90	1.94	1.87	1.76
Firmicutes	*Anaerotruncus colihominis*	2.00	2.10	2.01	2.02	1.98	2.24	2.14	2.07	2.00	2.30	2.20	2.25	1.85
Firmicutes	*Clostridium botulinum*	1.91	1.72	1.83	1.83	1.76	1.82	1.82	1.82	1.76	1.89	1.77	1.85	1.83
Proteobacteria	*Burkholderia multivorans*	1.76	1.68	1.67	1.69	1.84	1.91	1.96	1.83	1.81	1.91	1.69	1.75	1.67
Firmicutes	*Ruminococcus lactaris*	1.61	1.46	1.54	1.61	1.46	1.56	1.43	1.46	1.45	1.42	1.39	1.44	1.38
Firmicutes	*Clostridium leptum*	1.39	1.62	1.48	1.30	1.39	1.33	1.29	1.41	1.47	1.49	1.27	1.48	1.23
Firmicutes	*Bacillus megaterium*	1.23	1.35	1.23	1.16	1.17	1.19	1.18	1.11	1.18	1.17	1.36	1.29	1.02
Proteobacteria	*Salmonella enterica*	1.15	1.13	1.16	1.12	1.18	1.20	1.12	1.22	1.14	1.03	1.10	1.25	1.00
Bacteroidetes	*Bacteroides vulgatus*	1.12	1.03	1.00	1.02	1.10	1.07	1.17	1.12	1.02	1.05	1.11	1.14	1.18
Firmicutes	*Thermosinus carboxydivorans*	1.08	0.98	1.23	1.25	1.12	1.19	1.16	1.08	1.22	1.28	1.08	1.10	1.02
Bacteroidetes	*Bacteroides fragilis*	1.06	0.98	1.02	0.95	0.87	0.93	0.99	0.91	0.89	1.02	1.01	0.84	1.08

Supplementary Table 2. List of bacterial species identified based on contig based analysis. Percent abundance are shown here. Only above 1% are mentioned and sorted on Lib1.

**Phylum**	**Species**	**Lib1**	**Lib2**	**Lib3**	**Lib4**	**Lib5**	**Lib6**	**Lib7**	**Lib8**	**Lib9**	**Lib10**	**Lib11**	**Lib12**	**Lib-all**
Firmicutes	*Faecalibacterium prausnitzii*	7.26	6.31	6.15	7.29	6.91	8.02	6.22	6.67	7.25	6.32	6.84	6.96	6.41
Bacteroidetes	*Bacteroides spp.*	5.94	5.98	5.86	5.95	5.72	5.20	6.19	5.92	6.02	6.07	6.14	6.26	6.57
Firmicutes	*Ruminococcus spp.*	3.81	4.08	4.01	4.03	4.08	3.84	2.55	4.20	4.02	3.67	3.69	4.02	3.04
Firmicutes	*Ruminococcus sp.* 5_1_39BFAA	3.47	3.84	3.83	3.96	3.88	3.53	2.38	3.87	3.73	3.49	3.51	3.85	2.87
Firmicutes	*Coprococcus comes*	3.05	2.69	2.76	3.05	3.27	3.09	2.03	3.12	2.69	2.97	2.79	2.76	2.22
Bacteroidetes	*Bacteroides thetaiotaomicron*	3.00	3.10	3.47	3.02	3.49	2.97	2.91	3.45	3.33	3.15	3.63	3.06	3.30
Bacteroidetes	*Bacteroides ovatus*	2.66	2.73	2.91	2.33	2.56	2.72	2.69	2.54	2.60	2.43	2.49	2.52	2.51
Bacteroidetes	*Alistipes putredinis*	2.65	3.10	2.70	2.92	2.64	2.13	1.14	2.52	2.52	2.71	2.52	2.96	2.75
Firmicutes	*Dorea longicatena*	2.60	2.64	2.84	2.54	2.59	2.90	2.09	2.28	2.56	2.83	2.27	2.41	2.14
Bacteroidetes	*Bacteroides vulgatus*	2.51	2.61	2.47	2.95	3.02	2.66	1.98	3.10	2.99	2.51	3.11	2.92	3.18
Bacteroidetes	*Bacteroides fragilis*	2.31	2.28	2.26	1.99	2.08	2.09	2.61	2.52	2.05	2.22	1.76	2.16	2.06
Firmicutes	*Eubacterium rectale*	1.97	1.86	2.05	2.07	2.15	2.25	2.46	1.61	1.76	1.72	2.03	1.93	2.26
Bacteroidetes	*Parabacteroides distasonis*	1.76	2.16	2.06	2.20	1.90	1.69	1.52	1.63	1.79	2.16	2.24	2.02	2.36
Bacteroidetes	*Bacteroides helcogenes*	1.45	1.22	1.51	1.53	1.39	1.38	1.14	1.40	1.60	1.06	1.56	1.58	1.55
Bacteroidetes	*Bacteroides stercoris*	1.42	1.22	1.39	1.17	1.24	1.28	0.96	1.33	1.23	1.06	1.31	1.27	1.45
Bacteroidetes	*Bacteroides sp.* 3_1_40A	1.15	1.00	0.82	1.00	0.96	0.63	0.76	1.00	1.12	0.99	0.93	1.11	1.09
Bacteroidetes	*Bacteroides xylanisolvens*	1.04	0.89	1.02	0.90	0.79	0.93	0.97	0.98	0.83	0.99	0.93	0.87	0.77
Bacteroidetes	*Bacteroides sp.* 4_3_47FAA	1.01	0.85	0.74	0.82	1.06	0.57	0.71	1.07	0.99	0.78	0.87	1.10	1.08
Firmicutes	*Ruminococcus lactaris*	0.99	0.79	0.84	0.98	1.29	0.81	0.77	1.00	1.09	0.83	0.89	1.11	0.95

Supplementary Table 3. Random sampling of the metatranscriptome sequence reads and de-novo assembly of contigs.

Name	Read			Contig		
	Sequences (million)	Avg. size (bp)	Total bases (billion)	% of reads assembled in contig	Number of contig	Avg. size (bp)
HS100	30	100	3.0	98.5	8,253	213
MS151	30	144	4.3	98.9	42,153	200
MS301	30	178	5.4	98.3	97,631	313

Supplementary Table 4: Percent abundance of bacterial species in metatranscriptome data based on read and contig analysis.

Phylum	Species	HS100		MS151		MS301	
		Read	Contig	Read	Contig	Read	Contig
Firmicutes	*Faecalibacterium prausnitzii*	9.6	10.1	6.5	7.1	9.2	8.4
Bacteroidetes	*Bacteroides* spp.	4.4	3.9	6.3	5.0	3.5	4.7
Bacteroidetes	*Alistipes putredinis*	2.8	3.8	2.8	2.1	2.2	3.2
Bacteroidetes	*Bacteroides thetaiotaomicron*	2.0	2.7	3.2	2.4	1.8	2.7
Firmicutes	*Ruminococcus* spp.	2.7	4.4	3.4	3.0	2.1	2.5
Firmicutes	*Ruminococcus* sp. 5_1_39BFAA	2.6	4.2	3.2	2.9	2.1	2.4
Bacteroidetes	*Bacteroides vulgatus*	1.9	1.5	3.2	2.1	1.5	2.1
Firmicutes	*Dorea longicatena*	2.4	4.2	2.3	2.5	2.2	2.1
Firmicutes	*Coprococcus comes*	2.4	3.2	2.3	2.4	1.9	2.0
Firmicutes	*Eubacterium rectale*	1.3	2.0	2.2	1.6	1.3	2.0

Supplementary Table 5. List of accession numbers.

MG-RAST ID	
4589721.3	4681268.3
4589651.3	4681258.3
4589652.3	4681259.3
4594249.3	4681265.3
4594250.3	4681262.3
4594251.3	4681264.3
4594252.3	4681267.3
4594253.3	4681269.3
4594254.3	4681263.3
4589650.3	4681260.3
4589719.3	4681266.3
4589720.3	4681261.3
4637936.3	4681253.3
4589649.3	4681394.3
4671852.3	4671851.3
4681530.3	4638983.3
4681257.3	4681249.3
4681435.3	4681250.3
4681436.3	4681251.3

Supplementary Table 6. List of bacterial species/sequences identified in the metatranscriptomics data.

Sno	Firmicutes	% Abundance
1	Clostridium sp. RKD	0.0027
2	Thermoanaerobacter uzonensis	0.0014
3	Bacillus sp. F5	0.0014
4	Streptococcus sp. F.MI.5	0.0014
5	Streptococcus parauberis	0.0014

Sno	Bacteroidetes	% Abundance
1	Flexibacter elegans	0.0014
2	Porphyromonas cangingivalis	0.0014
3	Porphyromonas levii	0.0014

Sno	Actinobacteria	% Abundance
1	uncultured actinobacterium HF0500_35G12	0.0233
2	Streptomyces cinnamonensis	0.0014

Sno	Verrucomicrobia	% Abundance
1	uncultured Verrucomicrobiales bacterium HF0200_39L05	0.0014

Sno	Proteobacteria	% Abundance
1	Morganella morganii	0.0014
2	Pseudomonas denitrificans	0.0014
3	Pseudomonas oleovorans	0.0014
4	Pseudomonas sp. AW54a	0.0014
5	Pseudomonas sp. CT14	0.0014
6	Pseudomonas sp. ED23-33	0.0014
7	Pseudomonas sp. K-62	0.0027
8	Pseudomonas sp. K82	0.0014
9	Rickettsia akari	0.0014
10	Roseovarius nubinhibens	0.0069
11	Xanthomonas sp. W17	0.0014
12	Yersinia aldovae	0.0014
13	uncultured alpha proteobacterium HF0070_14E07	0.0041
14	uncultured alpha proteobacterium HF0130_06E21	0.0014
15	uncultured beta proteobacterium CBNPD1 BAC clone 578	0.0137
16	uncultured beta proteobacterium HF0010_04H24	0.0014
17	uncultured delta proteobacterium HF0200_14D13	0.0082
18	uncultured delta proteobacterium HF0200_19J16	0.0014
19	uncultured delta proteobacterium HF0200_39N20	0.0178
20	uncultured delta proteobacterium HF0770_45N15	0.0014
21	uncultured gamma proteobacterium EB750_07C09	0.0027
22	uncultured gamma proteobacterium HF0010_10D20	0.0014
23	uncultured Pseudomonadales bacterium HF0010_05E14	0.0014
24	uncultured Rhizobiales bacterium HF4000_32B18	0.0027
25	uncultured Rhodospirillales bacterium HF4000_24M03	0.0014

Supplementary Table 7. List of genes (Level 4 function) identified in MT dataset.

NEFL, NF-L; neurofilament light polypeptide
ACADM, acd; acyl-CoA dehydrogenase [EC:1.3.8.7]
cysI; sulfite reductase (NADPH) hemoprotein beta-component [EC:1.8.1.2]
cysJ; sulfite reductase (NADPH) flavoprotein alpha-component [EC:1.8.1.2]
cobC1, cobC; cobalamin biosynthetic protein CobC
GCDH, gcdH; glutaryl-CoA dehydrogenase [EC:1.3.8.6]
E1.4.1.9; leucine dehydrogenase [EC:1.4.1.9]
abmG; 2-aminobenzoate-CoA ligase [EC:6.2.1.32]
pdeA; c-di-GMP-specific phosphodiesterase [EC:3.1.4.52]
CTSL; cathepsin L [EC:3.4.22.15]
ARPC2; actin related protein 2/3 complex, subunit 2
lsrD; AI-2 transport system permease protein
COX15; cytochrome c oxidase assembly protein subunit 15
ttrC; tetrathionate reductase subunit C
desR; two-component system, NarL family, response regulator DesR
mprB; two-component system, OmpR family, sensor histidine kinase MprB [EC:2.7.13.3]
virB10, lvhB10; type IV secretion system protein VirB10
phoD; alkaline phosphatase D [EC:3.1.3.1]
ABC.NGC.P1; N-acetylglucosamine transport system permease protein
CYTB, petB; ubiquinol-cytochrome c reductase cytochrome b subunit
virB1; type IV secretion system protein VirB1
rpoA2; DNA-directed RNA polymerase subunit A'' [EC:2.7.7.6]
EIF5; translation initiation factor 5
EEF1A; elongation factor 1-alpha
poxA; lysyl-tRNA synthetase, class II [EC:6.1.1.6]
SRRM1, SRM160; serine/arginine repetitive matrix protein 1
E3.1.11.1, sbcB; exodeoxyribonuclease I [EC:3.1.11.1]
E3.4.15.1, ACE; peptidyl-dipeptidase A [EC:3.4.15.1]
ligJ; 4-oxalmesaconate hydratase [EC:4.2.1.83]
E1.8.2.1; sulfite dehydrogenase [EC:1.8.2.1]
HGD, hmgA; homogentisate 1,2-dioxygenase [EC:1.13.11.5]
agp; glucose-1-phosphatase [EC:3.1.3.10]
ligB; protocatechuate 4,5-dioxygenase, beta chain [EC:1.13.11.8]
E6.4.1.4A; 3-methylcrotonyl-CoA carboxylase alpha subunit [EC:6.4.1.4]
E3.1.1.17; gluconolactonase [EC:3.1.1.17]
E3.1.1.75, phaZ; poly(3-hydroxybutyrate) depolymerase [EC:3.1.1.75]
ubiH; 2-octaprenyl-6-methoxyphenol hydroxylase [EC:1.14.13.-]
PCCB, pccB; propionyl-CoA carboxylase beta chain [EC:6.4.1.3] - #1
E1.1.1.36, phbB; acetoacetyl-CoA reductase [EC:1.1.1.36]
atuG; citronellol/citronellal dehydrogenase
SCD, desC; stearoyl-CoA desaturase (delta-9 desaturase) [EC:1.14.19.1]

ligA; protocatechuate 4,5-dioxygenase, alpha chain [EC:1.13.11.8]
E5.1.1.8; 4-hydroxyproline epimerase [EC:5.1.1.8]
pduL; phosphotransacylase
mtnD, mtnZ, ADI1; 1,2-dihydroxy-3-keto-5-methylthiopentene dioxygenase [EC:1.13.11.53 1.13.11.54]
hcaC, bphF; dioxygenase ferredoxin subunit
cbiGH-cobJ; cobalt-precorrin 5A hydrolase / precorrin-3B C17-methyltransferase [EC:3.7.1.12 2.1.1.131]
ppx-gppA; exopolyphosphatase / guanosine-5'-triphosphate,3'-diphosphate pyrophosphatase [EC:3.6.1.11 3.6.1.40]
acnB; aconitate hydratase 2 / 2-methylisocitrate dehydratase [EC:4.2.1.3 4.2.1.99]
hpaE, hpcC; 5-carboxymethyl-2-hydroxymuconic-semialdehyde dehydrogenase [EC:1.2.1.60]
ligC; 2-hydroxy-4-carboxymuconate semialdehyde hemiacetal dehydrogenase [EC:1.1.1.312]
phhA, PAH; phenylalanine-4-hydroxylase [EC:1.14.16.1]
ACTA2; actin, aortic smooth muscle
